# Supplementary material for: Development of a computer-aided design software for the quantitative evaluation of aesthetic damage
Source: PLoS One. 2019 Dec 18;14(12):e0226322. doi: 10.1371/journal.pone.0226322 (PMC6919621; doi:10.1371/journal.pone.0226322)
Supplement: S1 Code site — 1. (ZIP) [file pone.0226322.s001.zip › [EESC_jr][Projeto]Relatorio_07_21/reports/5/2017.02.21/15:30:38/report.pdf]

Preâmbulo

Na data de 20/02/2017 no horário 13:30, o Perito(a) Mestranda Gabriela Cauduro da Rosa, Cirurgiã-dentista, designado(a)para proceder ao exame pericial solicitado em: Maria Silveira, do gênero Feminino, nascido(a) em 24/04/1985, ocupação Engenheira, portador(a) do RG 4097681541 SSP, cujo endereço Rua otacilio tomanik, passa a expor a avaliação de dano estético corporal.

Exame objetivo da lesão

A lesão ora objeto de análise teve como natureza Acidente pessoal ocorrido em 20/08/2016. O prejuízo estético consiste em lesão cicatricial consolidada na região: Rosto, Laterais (rosto), Lábios, Boca, Bochecha no lado esquerdo.

Trata-se de cicatriz Hipertrófica de tamanho/frequência Única: Retilínea de orientação Oblíqua de pigmentação Hipercrômica medindo 1 centímetro(s) na horizontal, 2.5 centímetros na vertical e 3 milímetros de Altura. De contornos Regulares. Perceptivel em 50 centímetros de distância nas relações íntimas. Perceptivel em 3 metros nas relações sociais. A lesão tem repercução na vida diária afetiva, social conforme fotos anteriores e atuais..

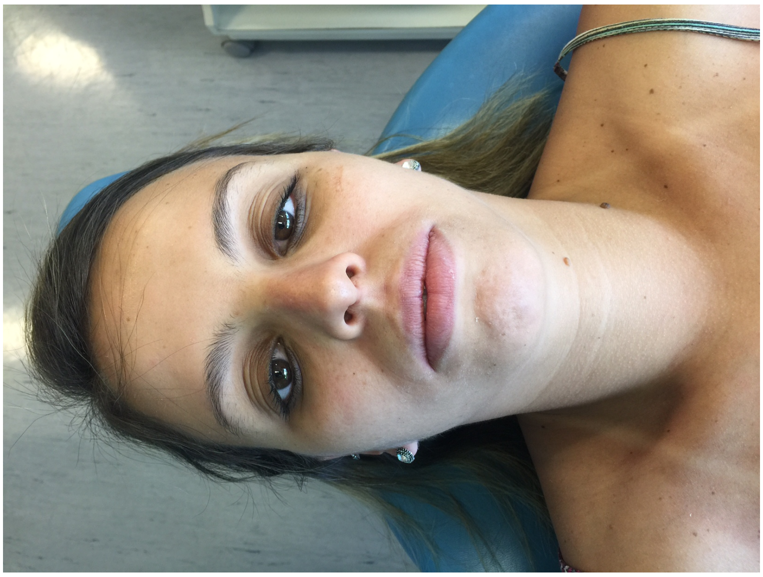

Conclui-se que classificada segundo os parâmetros de dano estético AIPE - Análise do Impacto do Prejuízo Estético (escala de seis níveis e pontuação entre 0 e 50 pontos), como sendo dano estético relativo à área 1, a lesão é caracterizada por 30 pontos e grau de prejuízo estético: Leve.

Escore final: 30;
Grau de prejuízo: Leve.

Santos, 20/02/2017

______________________________

Mestranda Gabriela Cauduro da Rosa
